# Molecular role of GATA binding protein 4 (GATA-4) in hyperglycemia-induced reduction of cardiac contractility

**DOI:** 10.1186/1475-2840-10-57

**Published:** 2011-06-24

**Authors:** Po-Ming Ku, Li-Jen Chen, Jia-ru Liang, Kai-Chun Cheng, Yin-Xiao Li, Juei-Tang Cheng

**Affiliations:** 1 Department of Cardiology, Chi-Mei Medical Center, No. 901 Chon-Hwa Road, Yong Kang, Tainan City, Taiwan; 2 Department of Medical Research, Chi-Mei Medical Center, No. 901 Chon-Hwa Road, Yong Kang, Tainan City, Taiwan; 3 Institute of Basic Medical Sciences, No.1 University Road, College of Medicine, National Cheng Kung University, Tainan City, Taiwan; 4 Department of Pharmacology, No.1 University Road, College of Medicine, National Cheng Kung University, Tainan City, Taiwan; 5 Department of Psychosomatic Internal Medicine, Kagoshima University Graduate School of Medical and Dental Sciences, 8-35-1 Sakuragaoka, Kagoshima City 890-8520, Japan

## Abstract

**Background:**

Diabetic cardiomyopathy, a diabetes-specific complication, refers to a disorder that eventually leads to left ventricular hypertrophy in addition to diastolic and systolic dysfunction. In recent studies, hyperglycemia-induced reactive oxygen species (ROS) in cardiomyocytes have been linked to diabetic cardiomyopathy. GATA binding protein 4 (GATA-4) regulates the expression of many cardio-structural genes including cardiac troponin-I (cTnI).

**Methods:**

Streptozotocin-induced diabetic rats and H9c2 embryonic rat cardiomyocytes treated with a high concentration of glucose (a D-glucose concentration of 30 mM was used and cells were cultured for 24 hr) were used to examine the effect of hyperglycemia on GATA-4 accumulation in the nucleus. cTnI expression was found to be linked to cardiac tonic dysfunction, and we evaluated the expression levels of cTnI and GATA-4 by Western blot analysis.

**Results:**

Cardiac output was lowered in STZ-induced diabetic rats. In addition, higher expressions of cardiac troponin I (cTnI) and phosphorylated GATA-4 were identified in these rats by Western blotting. The changes were reversed by treatment with insulin or phlorizin after correction of the blood sugar level. In H9c2 cells, ROS production owing to the high glucose concentration increased the expression of cTnI and GATA-4 phosphorylation. However, hyperglycemia failed to increase the expression of cTnI when GATA-4 was silenced by small interfering RNA (siRNA) in H9c2 cells. Otherwise, activation of ERK is known to be a signal for phosphorylation of serine105 in GATA-4 to increase the DNA binding ability of this transcription factor. Moreover, GSK3β could directly interact with GATA-4 to cause GATA-4 to be exported from the nucleus. GATA-4 nuclear translocation and GSK3β ser9 phosphorylation were both elevated by a high glucose concentration in H9c2 cells. These changes were reversed by tiron (ROS scavenger), PD98059 (MEK/ERK inhibitor), or siRNA of GATA-4. Cell contractility measurement also indicated that the high glucose concentration decreased the contractility of H9c2 cells, and this was reduced by siRNA of GATA-4.

**Conclusions:**

Hyperglycemia can cause systolic dysfunction and a higher expression of cTnI in cardiomyocytes through ROS, enhancing MEK/ERK-induced GATA-4 phosphorylation and accumulation in the cell nucleus.

## Background

Diabetes ranks among the main risk factors for the development of congestive heart failure (CHF) [1,2]. Many patients with CHF and hyperglycemic symptoms have accompanying abnormalities including obesity, dyslipidemia, and hypertension, which also lead to structural and functional abnormalities of the heart in cardiac dysfunction and CHF [3-6]. The pathogenesis of left ventricular diastolic dysfunction in diabetes and diabetic cardiomyopathy has been extensively studied [7]. Intramyocardial accumulation of triglycerides and extracellular deposition of excess collagen and advanced glycation products trigger glucolipotoxicity and activation of several signal pathways (insulin resistance, oxidative stress, renin-angiotensin system, adipokines, and inflammation) in a milieu of altered substrate metabolism [8,9].

Diabetic cardiomyopathy appears to be related to hyperglycemia. Reactive oxygen species (ROS) generation has been detected in cells exposed to a high glucose concentration. Cell death such as apoptosis plays a critical role in cardiac pathogenesis. Thus, hyperglycemia seems to be linked to apoptotic cell death in the myocardium *in vivo*. Actually, hyperglycemia-induced myocardial apoptosis is mediated by ROS produced owing to the high glucose concentration [10-12].

It has been indicated that mutations in the cardiac troponin I (cTnI) gene could lead to hypertrophic cardiomyopathy [13]. The proximal regions of the cardiac TnI gene regulate its specific expression in the heart. A proximal GATA-4-binding site in the cardiac TnI gene is necessary for the transcriptional activation of this gene *in vitro*, while other sites for GATA-4 DNA binding may contribute to the regulation of this gene [14]. Otherwise, it has been documented that MEK1-ERK1/2 signaling regulates the hypertrophic growth of cardiomyocytes through the transcription factor GATA-4 by direct phosphorylation of serine 105, which enhances DNA binding and transcriptional activation [15,16]. The ERK cascade plays an important role in the signaling pathway leading to the development of myocardial hypertrophy [17]. It is well-known that ROS can activate extracellular signal-regulated kinases (ERK1/2) [18]. Previous studies have indicated that ERK phosphorylation is important for the development of cardiac hypertrophy induced by hyperglycemia [17,19-21]. Otherwise, GSK3β has been described as an inhibitor of hypertrophic signaling in the intact myocardium [22]. GSK3β -induced nuclear export of GATA-4 may lower the nuclear accumulation of GATA-4, while inhibition of GSK3β by LiCl causes nuclear accumulation of GATA-4, suggesting that GSK3β negatively regulates the nuclear expression of GATA-4 [23].

Cardiac hypertrophy can be induced by hyperglycemia [24,25] and MEK/ERK signaling could be triggered in a high-glucose environment [26]. However, the role of GATA-4 in hyperglycemia-induced cardiac hypertrophy is still unknown. In the present study, we identified signals for hyperglycemia-induced cardiac hypertrophy, especially for GATA-4 accumulation in the nucleus, increasing cTnI over-expression.

## Methods

### Materials

Phlorizin was purchased from Fluka Chemie AG (Switzerland). Insulin (Monotard^® ^HM) was obtained from Novo Nordisk A/S (Bagsvaerd, Denmark). Tiron, PD98059 and SB203580 were obtained from Sigma Chemical Co. (St. Louis, MO, USA). Opti-MEM^® ^I Reduced Serum Medium, Stealth™ Select RNAi (siRNA-GATA-4), scrambled siRNA (siRNA-control), and Lipofectamine 2000™ were obtained from Invitrogen (Carlsbad, CA, USA). Antibodies of phospho-GATA-4 (serine105) (AB5245) and laminin (AB11575) were purchased from Abcam (Cambridge, UK). Antibodies for cardiac TnI (No. 4002), Phospho-GSK3β (Ser 9), GSK3β and Anti-rabbit IgG, HRP-linked were purchased from Cell Signaling Technology (Beverly, MA, USA). Antibodies to GATA-4, Histone H3 and goat anti-rabbit IgG-HRP-linked were obtained from Santa Cruz biotechnology, Inc. (Santa Cruz, CA, USA). Anti-Actin Monoclonal Antibody was purchased from Chemicon International, Inc. (Billerica, MA, USA). Fluorescent conjugated secondary DyLight™ 549-conjugated AffiniPure Donkey Anti-Goat IgG was purchased from Jackson ImmunoResearch Laboratories, Inc. (West Grove, PA, USA). 4', 6-diamidino-2-phenylindole (DAPI) was purchased from Thermo Fisher Scientific, Inc. (Rockford, IL, USA).

### Animal model

Eight-week-old male Wistar rats, weighing from 200 to 250 g, were obtained from the Animal Centre of National Cheng Kung University Medical College. Diabetes was induced by intravenous injection of 60 mg/kg streptozotocin as described previously[^1^]. Animals were considered hyperglycemic when their plasma glucose concentration was 20 mmol/L or greater in addition to polyuria and other hyperglycemic features. The levels of plasma glucose were measured in blood samples collected from the femoral vein of anesthetized rats (pentobarbital, 30 mg/kg, i.p.) one hour before treatment. Blood samples were centrifuged at 12,000 g for 3 min then analyzed using glucose kit reagents (AppliedBio assay kits; Hercules, CA, USA). The levels of plasma glucose were then estimated by an auto-analyzer (Quik-Lab, Ames, Miles, Inc., Elkhart, IN, USA), run in triplicate. All studies were carried out 10 weeks after induction of diabetes.

Then, STZ-diabetic rats were anesthetized with an intraperitoneal injection of urethane (1.4 g/kg) and cannulated in the right femoral artery with polyethylene catheters (PE-50). Mean arterial pressure (MAP) and heart rate (HR) were recorded using a polygraph (MP35, BIOPAC, CA, USA). The trachea was intubated for artificial ventilation (Small Animal Ventilator Model 683, Harvard Apparatus, Holliston, MA, USA) at 50 breaths/min with a tidal volume of 8 mL/kg and a positive end expiratory pressure of 5 cm H_2_O. After making an incision in the chest of the rat at the third intercostal space to expose the heart, a small section (1 cm long) of the ascending aorta was freed from the connective tissue. A Transonic Flowprobe (2.5PSB923, Transonic System Inc., Ithaca, NY, USA) was implanted around the root of the ascending aorta and connected to a transonic transit-time blood flowmeter (T403, Transonic System Inc.). The cardiac output (CO) was calculated from the aortic blood flow; the difference in CO between STZ-diabetic rats and normal rats was found.

Otherwise, 4 groups (n = 6 in each of groups) of age-matched rats were used for investigation: group I, vehicle-treated normal rats; group II, vehicle-treated STZ-diabetic rats; group III, insulin-treated STZ-diabetic rats; group IV, phlorizin- treated STZ-diabetic rats. The STZ-diabetic rats of group III received intraperitoneal (i.p.) injections of long-lasting human insulin (Monotards HM) at 22.75 μg/kg every 8 hr, three times per day, as described previously [27]. The STZ-diabetic rats of group IV received phlorizin by i.p. injection at a dose of 1 mg/kg every 8 hr according to the method described previously [27]. Phlorizin was dissolved in a 20% solution of propylene glycol and diluted with saline to the desired concentration for injection. Fluctuations in glycemia were not observed in rats that received repeated injections of insulin or phlorizin. Changes in protein expression were determined from samples collected after a 4-day treatment period. All animal procedures were performed according to the Guide for the Care and Use of Laboratory Animals published by the US National Institutes of Health (NIH Publication No. 85-23, revised 1996), as well as the guidelines of the Animal Welfare Act.

### Cell culture and treatment

Primary cultures of cardiomyocytes from neonatal rats were prepared with some modifications as described previously [1]. Briefly, heart tissue from a 1- to 2-day-old Wistar rat was cut into 1- to 2-mm pieces and predigested with trypsin to remove red blood cells. The heart tissue was then digested with 0.25% trypsin and 0.05% collagenase. The dissociated cells were placed in uncoated 10-cm dishes and incubated at 37°C in a 5% CO_2 _incubator for at least 1 hr to remove the nonmyocytic cells. This procedure caused most of the fibroblasts to attach to the dish, while most of the cardiomyocytes remained unattached. The population of cells enriched in cardiomyocytes was then collected and counted. The cells were cultured in Dulbecco's modified Eagle's medium (DMEM) purchased from GIBCO-BRL Life Technologies (GIBCO BRL, Gaithersburg, MD, USA) with 1 mmol/L pyruvate, 10% fetal bovine serum (FBS), 100 units/mL penicillin, and 100 units/mL streptomycin. On the second day after plating, the medium was replaced. Three days after plating, the cells were exposed to hyperglycemic conditions, as described in detail later. Animal handling and disposal were performed in accordance with the NIH guidelines. Embryonic rat-heart-derived H9c2 cells (BCRC No. 60096) were obtained from the Culture Collection and Research Center of the Food Industry Institute (Hsin-Chiu City, Taiwan). The H9c2 cells were maintained in growth medium composed of DMEM supplemented with 10% FBS. The H9c2 cells were plated at a density of 6000 cells/cm^2 ^and allowed to proliferate in growth medium. The medium was changed every 48 hr. To induce differentiation of the H9c2 myoblasts into myotubes, the growth medium was replaced by differentiation medium containing DMEM plus 2% horse serum when the cells neared confluence[28].

Hyperglycemia-treated cardiomyocytes were generated by treating the cells with 30 mmol/L glucose for 24 hr [1]. In brief, cells were supplemented with 10% FBS and antibiotics. After reaching approximately 60% confluence, serum-free cell media with various final concentrations of D-glucose (5.5, 10, 20 or 30 mM) were dispersed and cells were cultured for a further 24 hr [25]. We used H9c2 cells exposed to 5.5 mM D-glucose as a control. To rule out the effect of osmolarity, we added 24.5 mM mannitol to the control cultures. High glucose (HG)-treated cardiomyocytes were generated by incubating cells with 30 mmol/L glucose for 24 hr [25]. The cells were treated with tiron (100 nmol/L), PD98059 (20 μmol/L) and SB203580 (25 μmol/L) for 30 min before high-glucose treatment. After treating with a high glucose concentration for 24 hr, the cells were washed twice with PBS and removed by trypsinization. Cells were then collected for Western blot analysis.

### Measurement of superoxide generation

Superoxide production was analyzed by the lucigenin method with modification [29]. H9c2 cells (10^6 ^cells) were incubated in either control or HG medium for 24 hr in the presence or absence of the various treatments described above. We also treated cells with rotenone, a mitochondrial complex I inhibitor, as the positive control of superoxide generation [28,30]. H9c2 cells were then trypsinized, collected by centrifugation and washed. The resulting pellet was then re-suspended in 900 μL of Krebs buffer containing NaCl (130 mmol), KCl (5 mmol), MgCl_2 _(1 mmol), CaCl_2 _(1.5 mmol), K_2_HPO_4 _(1 mmol), and HEPES (20 mmol, pH 7.4, with 1 mg/mL bovine serum albumin (BSA). To measure ROS production, the suspensions were transferred into a measuring chamber and assessed using a Chemiluminescence Analyzer (Tohoku Electronic Industrial Co., Ltd., Japan). Suspensions were injected with 100 μL lucigenin (final concentration, 4 × 10^-4 ^mmol/L) and photon emissions were counted every 10 sec for up to 10 min.

### DHE staining

For intracellular ROS detection, H9c2 cells (about 5,000) were seeded onto 12-well cell culture plates. After 24 hr of growth, cultures were placed in either control or HG medium, treated as described above for 24 hr, and then harvested. Also, cells treated with rotenone were used as a positive control of superoxide generation [28,30]. The H9c2 cells were fixed with 4% paraformaldehyde for 30 min. After fixation, the cells were washed three times with PBS, and 5 μmol/L of dihydroethidium (DHE; Invitrogen) was added as a fluorescent indicator of ROS. Images were collected using an Olympus IX70 fluorescence microscope fitted with an Olympus America camera and MagnaFire 2.1 software.

### Immunofluorescence cell staining

H9c2 cells (about 10,000) were seeded onto 6-well cell culture plates. After 24 hr of growth, cultures were placed in either control or HG medium, treated as described above for 24 hr, and then harvested. The H9c2 cells were fixed with 4% paraformaldehyde for 30 min. After fixation, the cells were washed three times with phosphate-buffered saline (PBS). The tissue was blocked with 5% BSA for 30 min at room temperature. Primary antibodies diluted in 5% BSA (anti-phospho-GATA-4 1:100) were incubated at 4°C overnight. Thereafter, cells were washed in PBS and secondary antibodies (DyLight™ 549-conjugated AffiniPure Donkey Anti-Goat IgG 1:500) and were incubated for 30 min at room temperature. Cells were then washed with PBS and counterstained with 4', 6-diamidino-2-phenylindole (DAPI). Images were collected using an Olympus IX70 fluorescence microscope fitted with an Olympus America camera and MagnaFire 2.1 software.

### Nuclear protein extraction

For nuclear protein extraction, the CNMCS Compartmental Protein Extraction Kit was purchased from BioChain Institute, Inc. CA, USA. In briefly, Cells were collected using routine cell culture techniques. Count cells and add ice cold lysis buffer at 2.0 ml per 20 million cells. Mix the cells with buffer well and rotate the mixture at 4°C for 20 min. Syringe with a needle gauged between 26.5 and 30. Remove the needle tip by bending the needle several times and only leave the needle base on the syringe. Pass the cell mixture through needle base 50-90 times to disrupt the cell membrane and release the nuclei from the cells. The degree of cell membrane disruption and nucleus release can be monitored under microscope. Then spin the cells mixture at 15,000 g at 4°C for 20 min. The cytoplasmic proteins are in the supernatant, take out and save in another tube. Resuspend the pellet with ice cold wash buffer at 4.0 ml per 20 million cells; rotate at 4°C for 5 min. Spin at 15,000 g at 4°C for 20 min. Drain the supernatant. Add ice cold nuclear extraction buffer at 1.0 ml per 20 million cells to resuspend nuclear pellet, rotate at 4°C for 20 min then spin at 15,000 g at 4°C for 20 min. The nuclear proteins are in the supernatant, take out and save for further detections.

### Western blot analysis

Specific protein expression levels in rat hearts or H9c2 cells were determined by Western blot analysis. Protein was extracted from tissue homogenates and cell lysates using ice-cold **radio-immuno-precipitation assay (RIPA) **buffer supplemented with phosphatase and protease inhibitors (50 mmol/L sodium vanadate, 0.5 mM phenylmethylsulphonyl fluoride, 2 mg/mL aprotinin, and 0.5 mg/mL leupeptin). Proteins extracted using RIPA buffer were separated by sodium dodecyl sulfate polyacrylamide gel electrophoresis (SDS-PAGE), electrotransfered and immobilized on a nitrocellulose membrane. The membrane was blocked with 5% non-fat milk in PBS containing 0.1% Tween 20 (PBS-T) and incubated for 2 hr. The membrane was then washed in PBS-T and hybridized with primary antibodies, which were diluted to a suitable concentration in PBS-T, for 16 hr. Incubation with secondary antibodies and detection of the antigen-antibody complex were performed using an Enzymatic Chemiluminescence (ECL) kit (Amersham Biosciences, UK). Immunoblot densities were quantified using a laser densitometer.

### Small Interfering RNA (siRNA)

Duplexed RNA oligonucleotides for rat GATA-4 (Stealth RNAi ™) were synthesized by Invitrogen. H9c2 cells were transfected with 40 pmol of GATA-4-specific siRNAs (siRNA-GATA-4) or scramble siRNA (siRNA-control) using Lipofectamine 2000 (Invitrogen) according to the manufacturer's protocols that cells were treated with siRNA for 48 hr.

### Cell contractility assay

In the study of cell contraction, H9c2 cells were measured by the change in the planar surface area, as described previously [31]. H9c2 cells were cultured in 6-mm flat-bottomed plates approximately 5x10^5 ^cells/ plate. Culture plates were then mounted on the heated stage of an inverted light microscope for the contractility assay. Changes in the planar surface areas in response to treatment were observed using a video camera, and images of the cells were captured serially at 0, 20, 40, and 60 min. Cells of the same group (*n *= 6-10) were collected at the same time points. The perimeters of individual cells with clearly-defined borders were outlined, and the planar surface area was calculated as a percentage change. Control groups were monitored in cultures with vehicle alone.

### Statistical analysis

Data are expressed as the mean ± SEM for the number (n) of animals in one group as indicated. Statistical analysis was carried out using one-way ANOVA analysis and Newman-Keuls Post-hoc analysis. A *P*-value of 0.05 or less was considered significant.

## Results

### Effects of hyperglycemia on cardiac output, protein expression level of TnI and nucleus GATA-4 phosphorylation in diabetic rats

**Figure 1:**
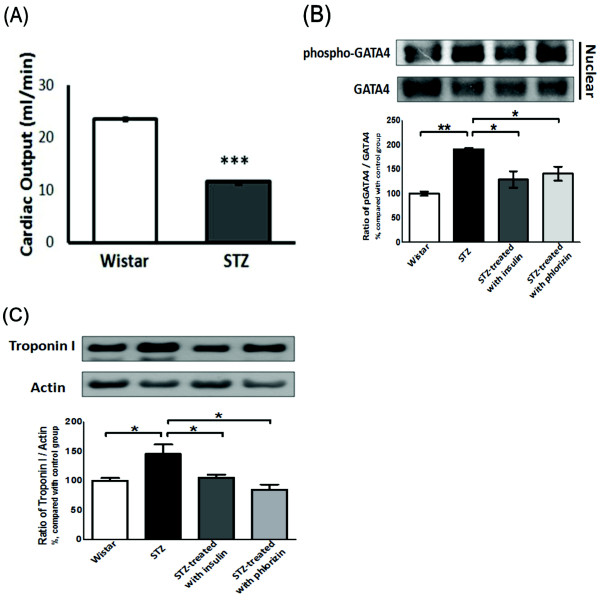
**Cardiac output and cardiac GATA-4 phosphorylation and troponin I expression in the heart**. Comparison of streptozotocin-induced diabetic rats (STZ) and normal (Wistar) rats. Each column shows the mean ± SEM (*n *= 6). ***P < 0.001 (A). STZ diabetic rats were treated with insulin or phlorizin at a dose sufficient to correct the blood sugar level for 4 days. Then, rats were sacrificed and nuclear GATA-4 phosphorylation (B) and troponin I expression (C) were detected by Western blot analysis. The results are presented as the mean ± SEM (*n *= 6 per group). *P < 0.05 and **P < 0.01 as compared with the STZ rats.

STZ-diabetic rats exhibited a lower cardiac output than the normal controls (23.6 ± 0.3 vs. 11.6 ± 0.4 ml/min, *P *< 0.001, *n *= 3) (Figure 1A). The levels of *TnI *protein expression and nucleus GATA-4 phosphorylation were significantly increased in the hearts of diabetic rats as compared with the normal controls (Figure 1B, 1C). After treatment with insulin or phloridzin for 7 days, in parallel to the reversion of blood sugar, recovery of *TnI *protein expression or nucleus GATA-4 phosphorylation was observed in the hearts of STZ-diabetic rats (Figure 1B, 1C).

### Characterization of superoxide generation in high-glucose-treated neonatal rat cardiomyocytes and H9c2 cells

**Figure 2:**
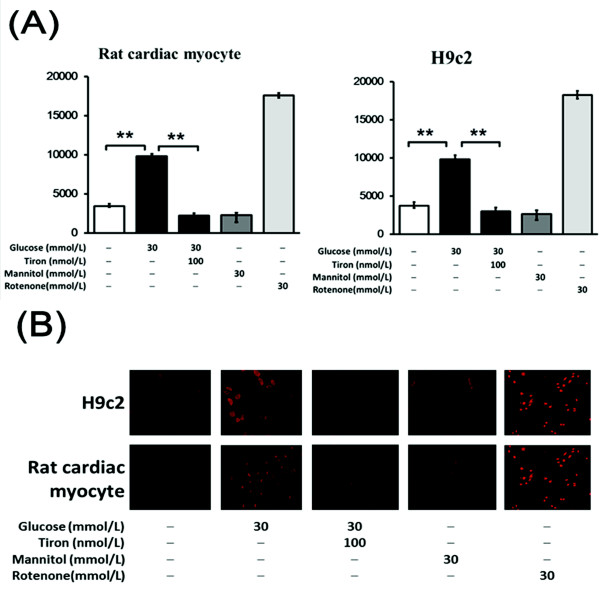
**Effects of tiron on ROS generation in neonatal rat cardiomyocytes and H9c2 cells**. DHE staining was used to visualize the intracellular ROS and lucigenin assays were used to quantify the generation of superoxide in neonatal rat cardiomyocytes (A) and H9c2 cells (B). *P < 0.05 and **P < 0.01 compared with the control; ^#^*P *< 0.05 and ^##^*P *< 0.01 compared with HG.

To understand the effect of a high-glucose environment on attenuating oxidative stress, we treated cells with various concentrations of D-glucose (5.5, 10, 20 or 30 mM). The lucigenin assays showed that the generation of intracellular ROS in HG-treated neonatal rat cardiomyocytes and H9c2 cells was enhanced as compared with their control groups (Figure 2). DHE staining also indicated that a high glucose concentration caused an increase in the superoxide level in both cultured neonatal rat cardiomyocytes (Figure 2A) and H9c2 cells (Figure 2B).

### Increase of TnI and phosphor-GATA-4 protein expression in high glucose-treated neonatal rat cardiomyocytes and H9c2 cells

**Figure 3:**
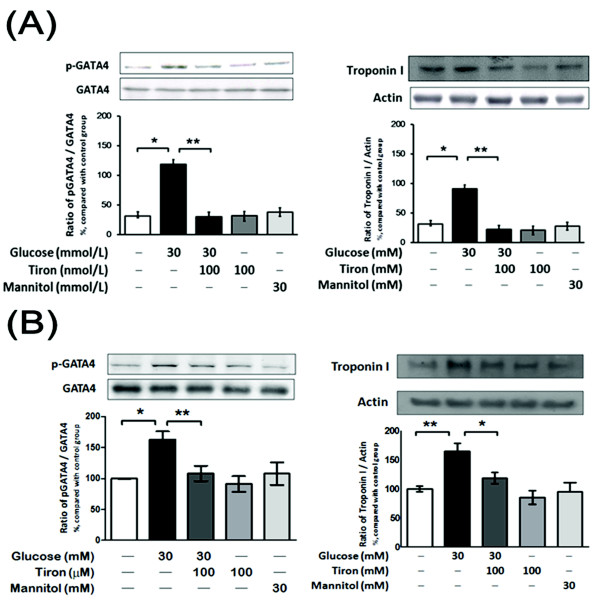
**GATA-4 and cardiac troponin I expression in neonatal rat cardiomyocytes and H9c2 cells**. Cell nuclear fractions were isolated for Western blot analysis to detect the phosphorylation level of GATA-4 and troponin I in neonatal rat cardiomyocytes (A) and H9c2 cells (B). All values are expressed as the mean ± SEM (*n *= 4). **P < 0.01 and *** *P *< 0.001 as compared with high glucose-treated H9c2 cells.

The effects of hyperglycemia on the protein levels of TnI and phospho-GATA-4 were further characterized in cultured neonatal rat cardiomyocytes and H9c2 cells exposed to various concentrations of glucose *in vitro*. The protein levels of TnI and phospho-GATA-4 in H9c2 cells were increased by high glucose concentrations after 24 hrs of incubation (Figure 3A, 3B). However, the expressions were not changed in mannitol-treated neonatal rat cardiomyocytes and H9c2 cells, indicating that mediation of hyperosmolarity seems unlikely. Actually, the increase in TnI or phospho-GATA-4 protein expression caused by a high glucose concentration in H9c2 cells is similar to that observed in diabetic hearts (Figure 1). This increase of TnI or phospho-GATA-4 protein expression in neonatal rat cardiomyocytes and H9c2 cells caused by a high glucose concentration was markedly reversed by tiron treatment (Figure 3). Thus, we continued our studies of H9c2 cells to investigate the possible mechanisms of HG-induced TnI and phosphor-GATA-4 protein expression in hyperglycemia-treated cells.

### Effect of mitogen-activated protein kinase (MAPK) on the phosphorylation of nucleus GATA-4 and expression of cTnI in high glucose-treated H9c2 cells

**Figure 4:**
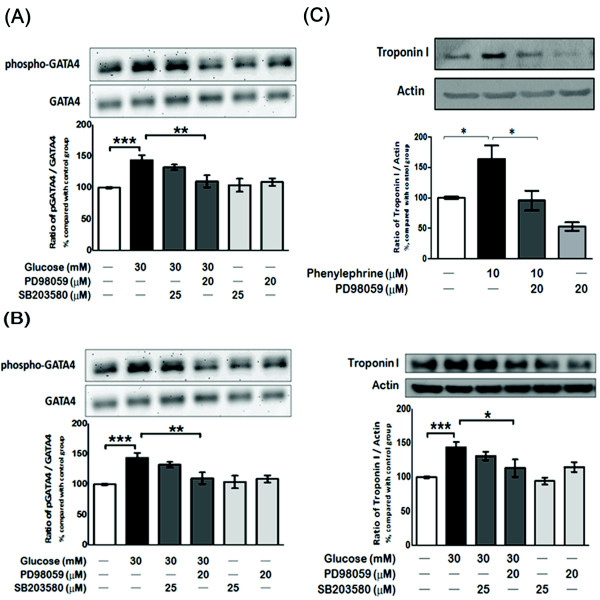
**Effects of MAPK inhibitors on the level of p-GATA-4 and cardiac cTnI expression**. H9c2 cells were incubated with SB203580 (25 mmol/L) or PD98059 (20 mmol/L) for 30 min before exposure to 30 mmol/L glucose. Then, nuclear fractions were isolated for Western blot analysis of GATA-4 phosphorylation (A) and cardiac troponin I expression (B). In another group, H9c2 cells were treated with 1 mmol/L phenylephrine (PE) in normal medium for 24 hr. Western blotting analysis was then used to estimate the effect of PD98059 on the expression of troponin I induced by PE (C). The results are presented as the mean ± SEM (*n *= 3-5 per group). *P < 0.05, **P < 0.01 as compared with the high glucose-treated group.

The transcriptional activity of GATA-4 could be enhanced via a p38 and MEK/ERK MAPK-dependent pathway to phosphorylate GATA-4 activation domains and GATA binding sites for the activation of target cardiac promoters [15,16,32]. To identify the role of MEK/ERK and the p38 MAPK-dependent pathway in GATA-4 phosphorylation and cTnI expression, PD98059 (20 μmol/L) (MEK/ERK inhibitor) and SB203580 (25 μmol/L) (p38 inhibitor) were used to treat H9c2 cells incubated in a high-glucose medium. We observed that PD98059 could reverse these actions (24%) but SB203580 failed to do so (Figure 4A and 4B). Furthermore, we treated H9c2 cells with phenylephrine (PE) as an activator of the MEK/ERK MAPK-dependent pathway, and found that the cTnI protein level expression was markedly increased in normal medium without high-glucose treatment. However, this change was also reversed by PD98059 (Figure 4C).

### Silencing of GATA-4 could reverse the expression of cTnI in high-glucose-treated H9c2 cells

**Figure 5:**
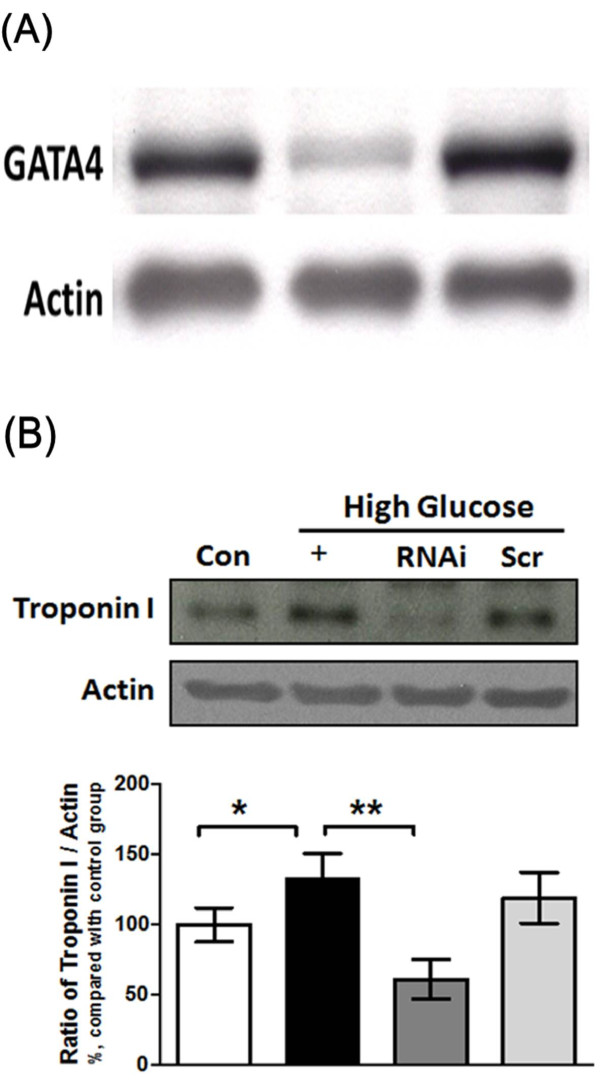
**Effects of GATA-4-specific RNAi on GATA-4 and cTnI expression in H9c2 cells**. H9c2 cells were transfected with siRNA specific to GATA-4 (RNAi) or scrambled RNA (Scr) for 24 hr and then exposed to 30 mmol/L glucose for another 24 hr. Whole cell lysates were isolated for Western blot analysis of GATA-4 (A) and troponin I (B) protein expression. H9c2 cells cultured in regular medium were used as the control (Con). The results are presented as the mean ± SEM (*n *= 3-5 per group). *P < 0.05, **P < 0.01 as compared with the high glucose-treated group.

We used siRNA specific for GATA-4 to silence the expression of GATA-4 (Figure 5A) and verified the GATA-4 induced cTnI generation in high-glucose-treated H9c2 cells. We found that the high-glucose-induced increase in cTnI expression was markedly reduced when GATA-4 was silenced (Figure 5B).

### GSK3β regulates nuclear localization of GATA-4 in neonatal rat cardiac myocytes and H9c2 cells

**Figure 6:**
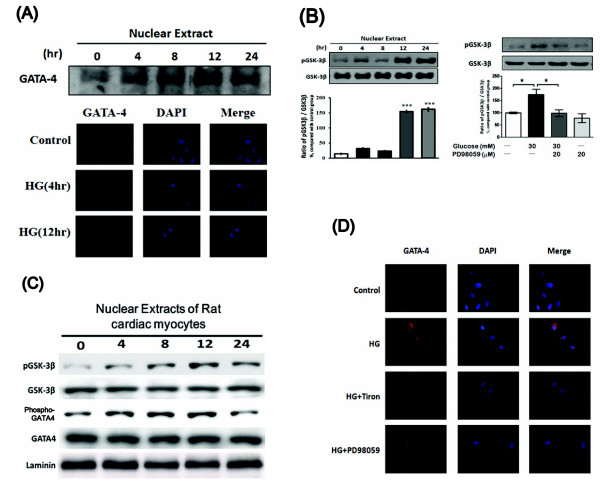
**Effect of MEK/ERK inhibitor on GSK3β phosphorylation and GATA-4 translocation in neonatal rat cardiacmyocytes and H9c2 cells**. H9c2 cells were exposed to 30 mmol/L glucose for 24 hrs, and subsequently the nuclear fraction and cytosolic fraction were isolated for Western blot analysis of GATA-4 expression and GSK3β phosphorylation (A and B). Changes in GATA-4 localization were also measured using a fluorescent microscope. Counter staining of the cell nucleus was achieved using DAPI (4', 6-diamidino-2-phenylindole) (A and D). H9c2 cells were incubated with PD98059 (20 mmol/L) for 30 min and subsequently exposed to the same glucose concentration (30 mmol/L) for 24 hr. The nuclear fractions were then isolated for Western blot analysis to measure phosphorylation of GSK3β (B). Result of western blot analysis in neonatal rat cardiomyocytes treated with HG at various doses(C). The results are presented as the mean ± SEM (*n *= 3 per group). *P < 0.05 as compared with the high glucose-treated group.

It has been established that GSK3β negatively regulates the nuclear expression of GATA-4 [23]. In the present study, GATA-4 was gradually increased in the nucleus by high-glucose treatment in a time-dependent manner (Figure 6A). Ser-9 phosphorylation of GSK3β has been reported to reverse GSK3β-induced inhibition of GATA-4 in cardiac myocytes [23]. Thus, we detected the level of GSK3β phosphorylation. The HG-induced phosphorylation level of GSK3β was increased in a time-dependent manner (Figure 6B). This change in GSK3β was correlated with the HG-induced nuclear translocation of GATA-4 (Figure 6A). Phosphorylation of GSK3β in H9c2 cells was significantly decreased by PD98059. Accumulation of GATA-4 in the nucleus was observed after GSK3β was inhibited by PD98059 (Figure 6B and 6D). Similar result was observed in neonatal cardiac myocytes (Figure 6C).

### The role of GATA-4 in H9c2 cell contraction

**Figure 7:**
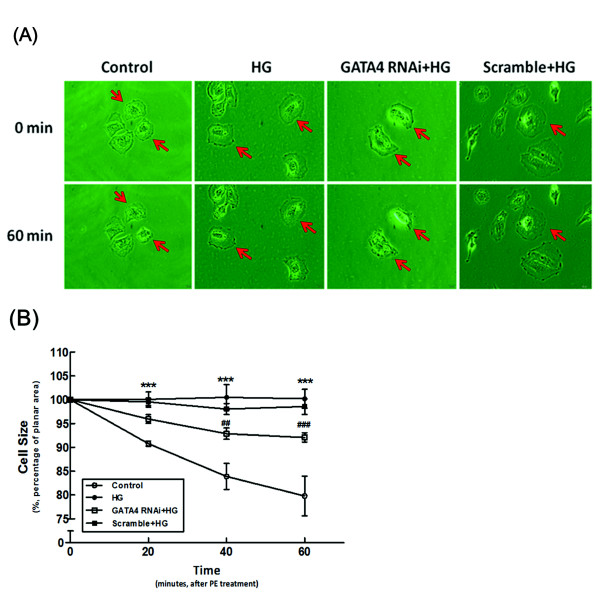
**Effects of GATA-4 specific siRNA on phenylephrine (PE)-induced cell contraction in H9c2 cells**. The high glucose-treated H9c2 cells were incubated with siRNA-GATA-4 (Si) or scrambled RNA as a control (Scramble) for 24 hr before PE challenge and then their planar surface area was measured. The upper panel (A) shows a representative change of cells in each group. The lower panel (B) expresses the differences between the four groups as the mean ± SEM (*n *= 6 per group). The high glucose-treated group differed markedly from the control incubated in normal medium (***P < 0.001). The high glucose-treated GATA-4 RNAi group differed markedly from the scrambled RNA-treated group treated with a high glucose concentration (^##^*P *< 0.01, ^###^*P *< 0.001).

The cell contraction assay was performed in the four experimental groups as follows: (1) in regular medium (control); (2) in high-glucose medium (HG); (3) incubation with siRNA of GATA-4 in high-glucose medium (GATA-4 RNAi + HG); (4) incubation with scramble RNA in high-glucose medium (Scramble + HG). Changes in the planar surface areas of cells in response to phenylephrine (PE) challenge at 0, 20, 40, and 60 min were recorded (Figure 7A). Before the addition of PE, the differences in the baseline planar areas of the four groups were statistically insignificant. After PE challenge, the cell planar areas decreased gradually and differed from their original sizes after 60 min. However, cells treated with a high glucose concentration lost contractility after PE challenge as compared with the control group (*P *< 0.05). H9c2 cells that received siRNA-GATA-4 in high-glucose medium exhibited no significant difference in cell contractility as compared with the control group. Loss of contraction was observed in the scramble RNA-treated cells under high-glucose treatment in a way significantly different (*P *< 0.05) from that of the siRNA of GATA-4-treated cells under high-glucose treatment (Figure 7B).

## Discussion

In the present study, we confirmed that diabetic rats exhibit a lower cardiac output than normal rats (Figure 1A). We also found that the expression of inhibitory troponin (cTnI) was significantly increased in the heart of diabetic rats (Figure 1C). Although diabetes-related cTnI expression has not been discussed previously, many studies have demonstrated that cTnI expression is markedly raised in other kinds of cardiac diseases [33-35] and drug-induced cardiac hypertrophy [36,37]. Similar to our previous report [38,39], correction of hyperglycemia by insulin or phlorizin reverses this higher expression of cTnI in STZ rats (Figure 1C). The role of hyperglycemia can thus be considered. In cultured neonatal rat cardiomyocytes and H9c2 cells, a cardiacmyocyte-like cell line, high-glucose treatment for 24 hr increased the expression of cTnI (Figure 3). The cell contractility was also significantly decreased by a high glucose concentration as compared with the control group in the phenylephrine challenge test (Figure 7). Both data collected from *in vitro *and *in vivo *experiments supported the major role of hyperglycemia.

It has been documented that high glucose concentrations may increase ROS production, causing diabetic cardiomyopathy [40]. Antioxidants could reverse high-glucose-induced cardiac injury [41-43]. In this study, tiron at concentrations sufficient to scavenge ROS also attenuated the expression of cTnI caused by high glucose concentrations. A role of intracellular ROS in the increased expression of cTnI can thus be considered in cardiac cells.

GATA-4 is found in cardiac myocytes and regulates many cardiac-specific gene expressions [44], such as atrial natriuretic factor (ANF), beta-type natriuretic peptide (BNP), myosin heavy chain (MHC), angiotensin II type IA receptor and endothelin-1. In adults, over-expression of GATA-4 could activate ventricular hypertrophic-related genes [45,46]. We observed that expression of GATA-4 is raised in STZ-diabetic rats (Figure 1B). It is of special interest that the ratio of phosphorylated GATA-4 and GATA-4 in the nucleus is changed with correction of the blood sugar level (Figure 1B). Moreover, the high glucose-induced increase in the expression of cTnI is reversed by siRNA of GATA-4 (Figure 5) in a way similar to the change in cell contractility (Figure 7). Thus, mediation of GATA-4 in the high glucose-induced increase in expression of cTnI is reliable.

It has been well-established that hypertension and stimulation with isoproterenol, phenylephrine, endothelin or other hypertrophic signals can activate GATA-4 through the MEK/ERK pathway to promote GATA-4 serine105 p38 phosphorylation for higher binding of GATA-4 on the specific promoter region to increase the transcriptional activity [15,16]. In the present study, PD98059 at a concentration (20 μmol/L) sufficient to inhibit MEK/ERK blocked the high glucose-induced GATA-4 serine105 phosphorylation and suppressed the expression of cTnI (Figure 4). We further used phenylephrine (10 μmol/L) to activate the MEK/ERK pathway in cells cultured in normal medium. Phenylephrine act as α-adrenergic receptor agonist that specifically activate MAPK(Mitogen-activated protein kinase) pathway without affect cAMP (cyclic adenosine monophosphate) activation that is induced by isoproterenol. The result showed that activation of the MEK/ERK pathway without high-glucose treatment could also increase the phosphorylation of GATA-4 serine105, as we have shown under high-glucose conditions previously. Furthermore, the results showed that phenylephrine could increase the expression of cTnI under normal conditions without a high glucose concentration (Figure 4). Cells treated with phenylephrine showed similar responses to high glucose treated group on GATA-4 to increase cTnI expression through MEK/ERK pathway can be considered.

Moreover, GATA-4-mediated transcription is markedly attenuated by GSK3β. GSK3β negatively regulates the nuclear expression of GATA-4 by stimulating nuclear export. Inhibition of GSK3β by α-adrenergic stimulation abrogates GSK3β-induced nuclear export of GATA-4, causing nuclear accumulation of GATA-4 [23]. In the present study, we observed that a high glucose concentration can increase the phosphorylation of GSK3β and GATA-4 accumulation in the nucleus. Also, both actions were blocked by PD98059, showing mediation of the MEK/ERK pathway.

The sacromere is constructed from thick filament and thin filament; the thick filament consists of myosin molecules and the thin filament is composed of actin, tropomyosin, and troponin complex. Transcriptional regulation of the MHC gene is complex and is initially activated by the intricate genetic program of cardiac development. However, GATA-4 depletion has been identified to show no effect on the expression of MHC [47]. In this study, we demonstrated that TnI is regulated by GATA-4. This finding may suggest that GATA-4 plays a role in modulation of the thin filament. Furthermore, it has been documented that high glucose is predominantly responsible for the decrease of IP3-induced Ca^2+ ^transients noted in VSMCs from diabetic animals [48]. Our findings indicated that GATA-4 attenuated the expression of TnI under hyperglycemic conditions. These results may use as one of the possible mechanisms to explain why cardiac contractility is decreased in rats suffering from hyperglycemia. However, the real mechanism(s) shall be investigated in the future.

## Conclusions

**Figure 8:**
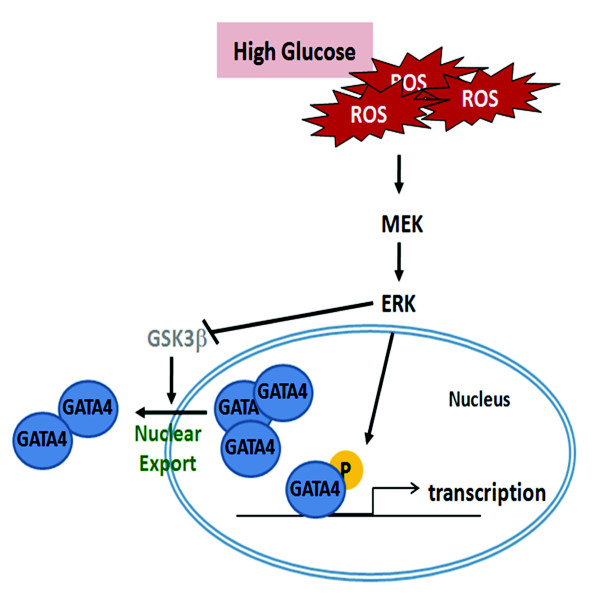
**Possible mechanism of hyperglycemia in change of cardiac troponin I expression in type-1 diabetic rats**. Hyperglycemia-induced ROS activates the MEK/ERK pathway to increase GATA-4 phosphorylation and nuclear translocation. Also, MEK/ERK activation causes GSK3β phosphorylation, lowering the export of GATA-4 and leading to GATA-4 preservation in the nucleus, which finally leads to an increase in the expression level of cTnI.

We have demonstrated that hyperglycemia-induced ROS activate the MEK/ERK pathway to increase GATA-4 phosphorylation for higher transcription. In addition, MEK/ERK activation causes GSK3β phosphorylation to lower the export of GATA-4 and leads to further GATA-4 accumulation in the nucleus. Thus, the expression of cTnI is raised (Figure 8). Taken together, GATA-4 activation and nuclear translocation are important factors that lead to a reduction in cardiac contractility in diabetic cardiomyopathy.

## List of abbreviations

ROS: reactive oxygen species; GATA-4: GATA binding protein 4; cTnI: cardiac troponin-I; siRNA: small interfering RNA; GSK3β: glycogen synthase kinase 3 beta; CHF: congestive heart failure; ERK1/2: activate extracellular signal-regulated kinases; MAP: mean arterial pressure; HR: heart rate; STZ: streptozotoxin; CO: cardiac output; i.p.: intraperitoneal; DHE: dihydroethidium; HG: high glucose; PBS: phosphate-buffered saline; BSA: bovine serum albumin; DAPI: 4', 6-diamidino-2-phenylindole; RIPA: radioimmunoprecipitation assay; SDS-PAGE: sodium dodecyl sulfate polyacry lamide gel electrophoresis; ECL: enzymatic chemiluminescence; PE: phenylephrine; MAPK: mitogen-activated protein kinases; ANF: atrial natriuretic factor; BNP: beta-type natriuretic peptide; MHC: myosin heavy chain; SD: standard deviation; SEM: standard error of mean.

## Competing interests

The authors declare that they have no competing interests.

## Authors' contributions

JTC, LJC and JRL carried out the molecular studies and drafted the manuscript. KCC and YXL were involved in the interpretation of the results. JTC and PMK conceived the study and participated in its design, interpretation and coordination, and drafted and approved the manuscript. All authors have read and approved the final manuscript. Also, all authors contributed significantly to and are in agreement with the content of the manuscript.
